# An expressed sequence tag (EST) library from developing fruits of an Hawaiian endemic mint (*Stenogyne rugosa*, Lamiaceae): characterization and microsatellite markers

**DOI:** 10.1186/1471-2229-6-16

**Published:** 2006-08-23

**Authors:** Charlotte Lindqvist, Anne-Cathrine Scheen, Mi-Jeong Yoo, Paris Grey, David G Oppenheimer, James H Leebens-Mack, Douglas E Soltis, Pamela S Soltis, Victor A Albert

**Affiliations:** 1 Natural History Museum, University of Oslo, PO Box 1172 Blindern, 0318 Oslo, Norway; 2 Department of Botany, University of Florida, Gainesville, FL 32611, USA; 3 Department of Biology and Huck Institutes of the Life Sciences, The Pennsylvania State University, University Park, PA 16802, USA; 4 Florida Museum of Natural History, University of Florida, Gainesville, FL 32611, USA

## Abstract

**Background:**

The endemic Hawaiian mints represent a major island radiation that likely originated from hybridization between two North American polyploid lineages. In contrast with the extensive morphological and ecological diversity among taxa, ribosomal DNA sequence variation has been found to be remarkably low. In the past few years, expressed sequence tag (EST) projects on plant species have generated a vast amount of publicly available sequence data that can be mined for simple sequence repeats (SSRs). However, these EST projects have largely focused on crop or otherwise economically important plants, and so far only few studies have been published on the use of intragenic SSRs in natural plant populations. We constructed an EST library from developing fleshy nutlets of *Stenogyne rugosa *principally to identify genetic markers for the Hawaiian endemic mints.

**Results:**

The *Stenogyne *fruit EST library consisted of 628 unique transcripts derived from 942 high quality ESTs, with 68% of unigenes matching *Arabidopsis *genes. Relative frequencies of Gene Ontology functional categories were broadly representative of the *Arabidopsis *proteome. Many unigenes were identified as putative homologs of genes that are active during plant reproductive development. A comparison between unigenes from *Stenogyne *and tomato (both asterid angiosperms) revealed many homologs that may be relevant for fruit development. Among the 628 unigenes, a total of 44 potentially useful microsatellite loci were predicted. Several of these were successfully tested for cross-transferability to other Hawaiian mint species, and at least five of these demonstrated interesting patterns of polymorphism across a large sample of Hawaiian mints as well as close North American relatives in the genus *Stachys*.

**Conclusion:**

Analysis of this relatively small EST library illustrated a broad GO functional representation. Many unigenes could be annotated to involvement in reproductive development. Furthermore, first tests of microsatellite primer pairs have proven promising for the use of *Stenogyne rugosa *EST SSRs for evolutionary and phylogeographic studies of the Hawaiian endemic mints and their close relatives. Given that allelic repeat length variation in developmental genes of other organisms has been linked with morphological evolution, these SSRs may also prove useful for analyses of phenotypic differences among Hawaiian mints.

## Background

**Figure 1:**
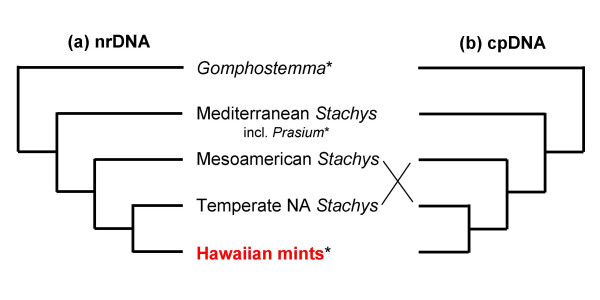
**Simplified phylogenetic tree of the Hawaiian mint relationships**. Phylogenetic analyses based on (a) 5S-NTS nuclear ribosomal DNA (nrDNA) and (b) chloroplast DNA (cpDNA) show the position of the endemic Hawaiian mints within the *Stachys *lineage [3]. The cross, likely indicative of an interlineage reticulation event [3], shows the different positions of Mesoamerican and temperate North American *Stachys *species with respect to one another and the Hawaiian mints. Taxa marked with an asterix (*) are those with fleshy nutlets (excluding the Hawaiian genus *Haplostachys*). These, including the monotypic genus *Bostrychanthera*, are the only taxa with fleshy fruits in the entire subfamily Lamioideae.

It has frequently been noted that many island plant lineages show little genetic sequence divergence compared with their continental relatives [e.g., [1,2]]. This phenomenon can be associated with recent radiations and/or extensive gene flow, and in the case of young plant lineages, as in the Hawaiian Islands, it is often contrasted with considerable morphological and ecological diversity. An example is the native Hawaiian mints (Lamiaceae), which represent a major island radiation that likely originated from polyploid ancestors in North American *Stachys *(Fig. 1) [3]. *Stachys *is a large (ca. 300 species) and widespread genus belonging to the subfamily Lamioideae. Based on sequence variation in the rapidly-evolving nuclear ribosomal 5S non-transcribed spacer (5S-NTS), the Hawaiian mints are most closely related to temperate North American *Stachys*, whereas with chloroplast (cpDNA) sequence data, they group with a largely Mesoamerican *Stachys *lineage (see Fig. 1). This phylogenetic incongruence between nuclear and organellar DNA data probably indicates a reticulate ancestry for the Hawaiian mints. Their closest relatives appear to be bird-pollinated *Stachys chamissonis *and insect-pollinated *S. quercetorum*.

The Hawaiian mints comprise a total of 58 species in three genera. Dry-fruited *Haplostachys *and fleshy-fruited *Phyllostegia *and *Stenogyne *exhibit broad morphological and ecological variation. The sole extant member of *Haplostachys *(a genus of 5 species total), *H. haplostachya*, has fragrant white flowers typical of insect-pollination and is found in the xerophytic shrubland of Hawai'i. The flowers of *Phyllostegia *resemble those of *Haplostachys*. The 32 species of Hawaiian *Phyllostegia *are herbs, lianas or subshrubs and are mainly found in mesic to wet forest habitats. In *Stenogyne *the flowers are axillary and the tubular corollas, displaying a range of sizes and colors, usually have a reduced lower lip, suggestive of bird-pollination. The 21 species of *Stenogyne *are mainly perennial vines found in lower elevation, mesic-wet forests to higher elevation, subalpine woodland. In contrast to this extensive morphological and ecological diversity among the Hawaiian taxa, DNA sequence variation has been found to be remarkably low, resulting in a lack of phylogenetic resolution among accessions of *Phyllostegia *and *Stenogyne *[4]. It is apparent that faster evolving markers are necessary to study the presumed hybrid origin and adaptive evolutionary aspects, such as development of fleshy fruits, of the Hawaiian endemic mints.

In the search for suitable molecular markers to elucidate recent radiations, non-coding regions of chloroplast and nuclear gene sequences and sometimes variation within DNA fragment data, e.g., from restriction fragment length polymorphism (RFLP), random amplified polymorphic DNA (RAPD), and amplified fragment length polymorphism (AFLP), have been used in a diverse array of plants. Genomic microsatellites or simple sequence repeats (SSRs) have only had limited use for studies of natural plant populations, since they have to be developed each time in different plant species, which can be time consuming and expensive. However, among the advantages of microsatellite markers are their high reproducibility, multiallelic nature, codominant inheritance, relative abundance, that they usually have good genome coverage, and that only a small amount of DNA template is required [5].

In the past few years, expressed sequence tag (EST) projects have generated a vast amount of publicly available sequence data from plant species. These data are not only useful for gene discovery and comparative genomic investigation of transcriptomes and certain developmental processes, but they can also be mined for SSRs (typically referred to as EST or intragenic SSRs). These EST SSRs are useful as molecular markers in plant genetic and evolutionary studies because (i) they represent transcribed genes, (ii) a putative function can often be deduced by a homology search, and (iii) since they are derived from transcripts, they are useful for assaying functional diversity in natural populations [6]. Another important feature of EST-SSR markers is their expected higher levels of transferability to related species than genomic SSR markers. Several studies have now demonstrated not only high rates of infra-generic transferability but also transferability to other closely related genera [see [6]], which is also very promising for comparative mapping and genomic investigations of natural populations. However, EST projects have largely focused on crop or otherwise economically important plants, although EST data from other plants are emerging [7-9]. One example is the Floral Genome Project [10], which as part of a comparative genomic investigation of the floral transcriptome, has constructed a large set of ESTs from phylogenetically basal angiosperms specifically selected to bridge the evolutionary gaps between model plants [11].

**Figure 2:**
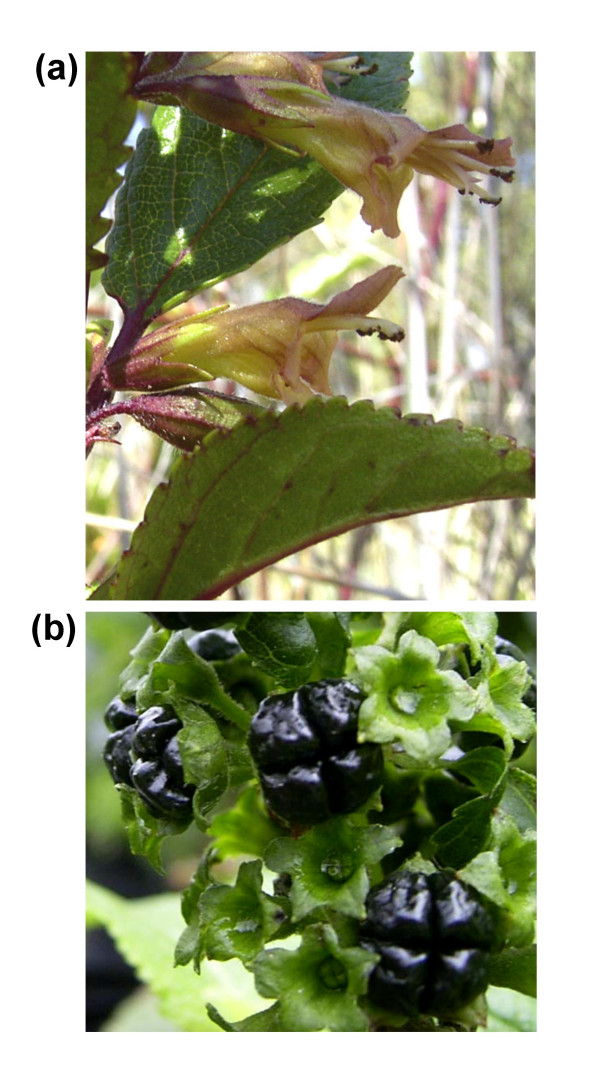
**Flowers and fruits of Hawaiian mints**. (a) *Stenogyne rugosa *flowers, and (b) fruits of *Phyllostegia ambigua *comprising four fleshy nutlets.

We constructed an EST library from developing fleshy fruits (nutlets) of *Stenogyne rugosa *(Fig. 2). Fleshy fruits are rare in the mint family and are only found in few other genera within the entire subfamily Lamioideae (Fig. 1). However, studies of fruit pericarp structure have shown that the fleshy nutlets of the Hawaiian mints are not anatomically homologous to other fleshy-fruited genera in the subfamily [12]. It is possible that fixation of this novel feature after the colonization of the Hawaiian Islands along with sorting out of floral morphotypes ensuring greater pollinator specialization has had a major adaptive impact on the evolution of the Hawaiian mint lineage [4].

By constructing a Hawaiian mint fruit EST library we primarily wished to search for and develop genetic markers for our Hawaiian endemic mint research. Furthermore, we wished to enable comparative reproductive transcriptome studies, e.g., with unigene sets from the Floral Genome Project and tomato (also an asterid angiosperm) [13]. In this paper we describe the EST library and show how a relatively small fruit EST data set can cover a broad functional representation of the general angiosperm transcriptome. Furthermore, we discuss several potential uses of this database, in particular as related to our search for genetic markers.

## Results

### EST library characterization

Average insert size in our cDNA library was 1500 bp. Random 5' sequencing of cDNAs from developing fruits generated a total of 1273 ESTs, resulting in 942 sequences (74%) passing quality check (see Table 1). Assembling the EST sequences into contigs gave a total of 628 unique gene sequences (unigenes), consisting of 352 singletons (56%) and 276 assemblies, most of which (92%) contained two ESTs per contig. The unigenes had an average length of 480 base pairs. Each unigene was given a numeric identifier (260504-261131). The EST data is available through the Plant Genome Network (PGN) website [14] as well as GenBank.

### Annotation and functional classification of unigenes

**Figure 3:**
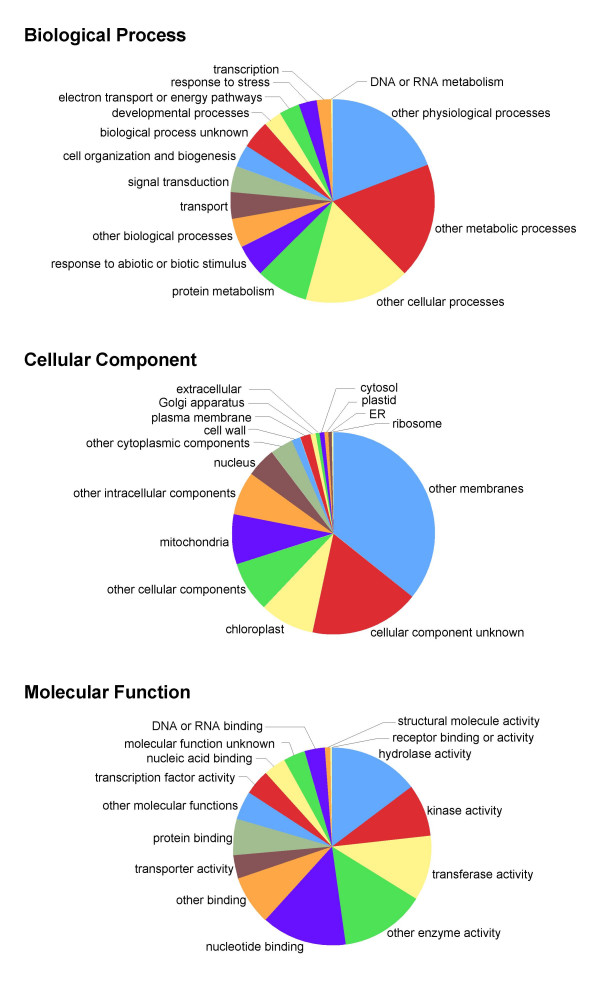
**Gene Ontology (GO) classification of the *Stenogyne *EST library**. The relative frequencies of GO hits for *Stenogyne rugosa *unigenes assigned to the GO functional categories Biological Process, Molecular Function, and Cellular Component, as defined for the *Arabidopsis *proteome.

**Figure 4:**
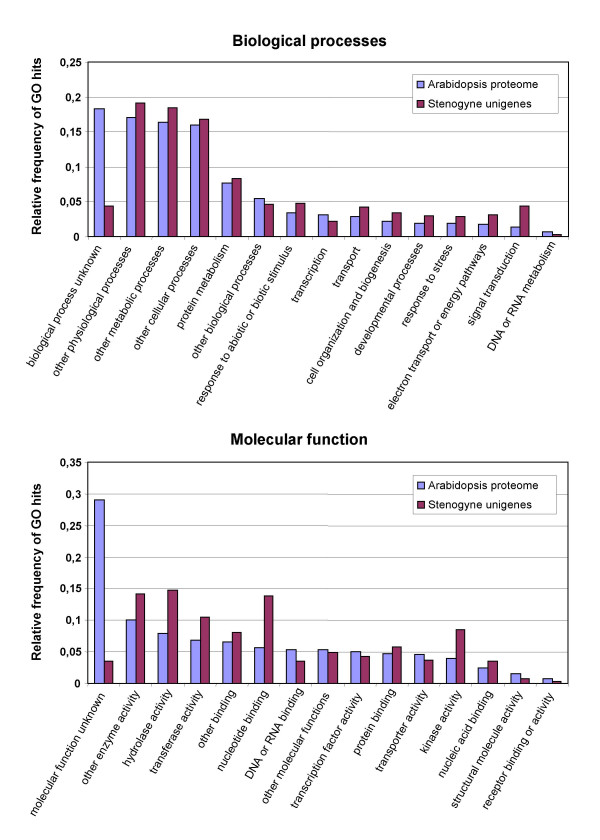
**Comparison of GO classification frequences for *Stenogyne *unigenes and the inferred *Arabidopsis *proteome**. Relative frequences assigned to the GO categories Biological Processes and Molecular Function are shown.

Using BLASTX to annotate the *Stenogyne *unigene sequences, 68% of unigenes matched *Arabidopsis *genes with an expectation value of 1e-10 or better. To get a better overview of the annotated unigenes, we used the Gene Ontology (GO) annotation [15] search on the TAIR website [16] to classify the unigenes into functional category. Relative frequencies of GO hits for *Stenogyne rugosa *unigenes assigned to the functional categories Biological Process, Molecular Function, and Cellular Component (as defined for the *Arabidopsis *proteome) are presented in Fig. 3. Comparison of frequencies with *Arabidopsis *(using whole *Arabidopsis *genome annotation on the TAIR website) within the Biological Processes and Molecular Function GO categories showed overall consistency in terms of representation within the functional classes (Fig. 4). Some of the unigenes could be identified as putative homologs of genes that are active during plant reproductive development (Table 2).

### Comparison with a tomato unigene set

The *Stenogyne *fruit EST library was compared with tomato unigene sets to identify genes essential for development, fruit development in particular. First, *Stenogyne *and tomato unigenes (downloaded from the Solanaceae Genomics Network; [13,17]) were annotated by best-matched hits (BLASTX e-value < 1e-10) to the *Arabidopsis *genome giving a total of 3276 and 12648 hits, respectively. Among these, 1745 hits were shared between *Stenogyne *and tomato. Using the GO Slim functional classification scheme [15,18], 121 loci were identified within Developmental Processes. As a result, many genes shared between the *Stenogyne *and tomato unigene sets may be relevant for fruit/embryo/seed development.

### Microsatellite discovery and preliminary results

A total of 42 unigenes (7%), comprising ca. 5% of the total EST sequences, contained 44 potentially useful microsatellite loci. Two of the unigenes contained two microsatellite loci each. Trinucleotide repeats were the most abundant (>50%) followed by tetra- (16%), penta- (16%), and dinucleotide (11%) repeats (see Table 3). Sequences for nine SSRs could be annotated to transcription factor or other nucleotide binding activity. Seven SSRs could be annotated to developmental processes (including the flowering time gene *FCA*).

**Figure 5:**
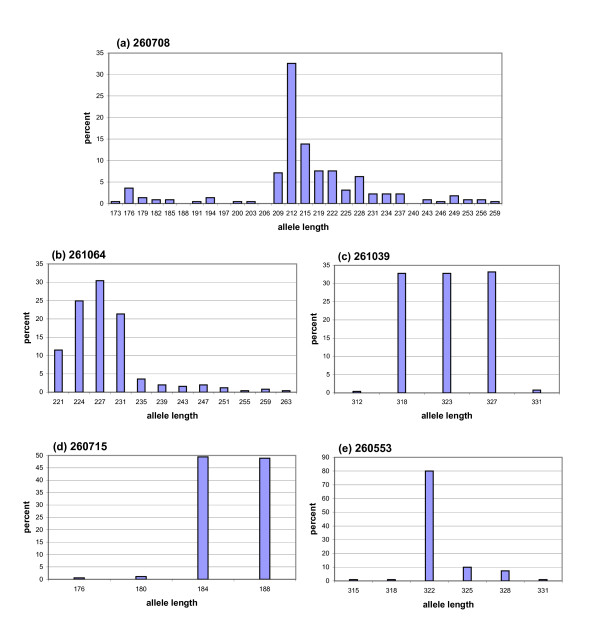
**Allele frequency distributions of five EST SSR markers**. SSR markers (a) 260708, (b) 261064, (c) 261039, (d) 260715, and (e) 260553. See also Tables 3–5 for more information on these loci.

Of the 35 SSR primer sets initially evaluated with homologous genomic DNA from *Stenogyne rugosa*, 30 primers gave clear PCR bands within the approximate range of expected product size. These were further tested for cross-transferability to other Hawaiian mints using the species *Stenogyne calaminthoides*, *Phyllostegia warshaueri*, and *Haplostachys haplostachya*. Amplification gave positive results for 24 primer pairs in all four taxa (see Table 4), and five primer pairs were further tested for variation among a total of 88 individual accessions, including 84 Hawaiian mint accessions and four North American relatives in the genus *Stachys*. Results from these five SSR loci are presented in Table 5 and Fig. 5. Two of the five loci proved to be highly variable, with a total number of alleles of 12 and 25, respectively, whereas the three remaining loci showed relative homogeneity among 1, 2 or 3 alleles, with some rare alleles also present (Fig. 5).

## Discussion

The Hawaiian endemic mints represent one of the largest plant lineages in the archipelago, exhibiting extensive vegetative, habit and reproductive diversity. Targeting single genes and comparing DNA sequences of already developed phylogenetic markers have proven very limited for understanding the molecular basis for this fascinating morphological radiation. Recent efforts have demonstrated that expressed sequence tag (EST) sequencing can be used as an efficient and relatively economical approach for large-scale gene discovery and comparative genomics research. Great potential for addressing many questions in evolutionary biology will be afforded as more data from non-model organisms emerge and are mined for functional information. We constructed a fruit EST library from an Hawaiian endemic mint as a further step toward understanding the molecular basis for the radiative evolution of the Hawaiian mint lineage and to provide resources for comparative genomics investigations of plant reproductive development.

### EST library annotation

A relatively high frequency (68%) of *Arabidopsis *genes were identified with our small fruit EST library. Also, analysis of GO annotations for the *Stenogyne *unigenes showed a fairly consistent sampling of functional classes as defined for the *Arabidopsis *proteome (Fig. 3). As similarly discovered by the Floral Genome Project [11], this suggests that a limited EST data set can provide a broad functional representation relative to the entire *Arabidopsis *transcriptome. For example, within the GO categories Biological Processes and Molecular Function, relatively even representation of the different functional classes is evident between the *Stenogyne *unigene set and the inferred *Arabidopsis *proteome (Fig. 4). In Biological Processes, the three most represented functional classes for the two transcriptomes are 'other physiological processes', 'other metabolic processes', and 'other cellular processes'. In the case of Molecular Function, the predominant functional classes belong to enzyme activity. The class 'nucleotide binding' is particularly well represented in the *Stenogyne *EST set. Unknown processes or functions are underrepresented in our EST data set relative to *Arabidopsis*. This was also seen with the comparison of the Floral Genome Project EST sets to the *Arabidopsis *proteome [see Fig. 2 in [11]]. One possible reason for this result is that many, perhaps rare, unidentified transcripts from the entire *Arabidopsis *proteome are not discovered using an EST library approach.

Concerning our 'positive' unigene identifications, it should be borne in mind that BLAST-based annotation of short EST unigenes can be misleading if best BLAST hits are more representative of domain homologies rather than homology to particular orthologs. However, at least among the five loci for which we analyzed microsatellite variation in depth, the four with putative counterparts in *Arabidopsis *could be annotated to specific orthologs in the *Arabidopsis *genome (see below).

### Reproductive developmental genes

Some of the unigenes from our EST library could be identified as putative homologs of *Arabidopsis *genes that are active during plant reproductive development (Table 2). Among these are *ABNORMAL FLORAL ORGANS *(*AFO*), *ARABIDOPSIS THALIANA CENTRORADIALIS *(*ATC*), *FCA*, *GIGANTEA *(*GI*), and *LUMINIDEPENDENS *(*LD*).

*AFO*, which has also been named *FILAMENTOUS FLOWER *(*FIL*) or *YABBY1*, belongs to a small family of genes encoding transcription factors. The primary function of the *YABBY *gene family members is to specify abaxial cell fates in lateral organs produced by apical and flower meristems [19]. Homologs of *YABBY *gene family members have also been found in many other EST library sets, e.g., in developing tomato fruits [20], developing reproductive tissues of a variety of basal angiosperms [11], and in young cycad leaves [7]. A microsatellite repeat found within our *AFO *homolog was analyzed for variation among a large number of individual accessions (see below).

*FCA*, *GI*, and *LD *are flowering time genes. Flowering time control is a complex process and molecular genetic analyses in *Arabidopsis *have identified many genes, as well as a variety of environmental factors, that regulate the transition to flowering [e.g., [21]]. Interestingly, although *FCA*, *GI*, and *LD *are genes that promote flowering, EST homologs are found expressed in developing tomato fruits [20]. *ATC *belongs to a small gene family that also includes *TERMINAL FLOWER1 *(*TFL1*), a key gene in regulating flowering time and maintaining the inflorescence meristem [e.g., [22-24]]. If *ATC *acts similarly to *TFL1 *it would be a flowering inhibitor. Whereas *TFL1 *is expressed in both the inflorescence meristem and during the vegetative phase, *ATC *was found to be expressed in the hypocotyl of young *Arabidopsis *plants [25]. Although overexpression experiments with *ATC *showed a phenotype similarly to constitutively expressed *TFL1*, it was suggested that *ATC *and *TFL1 *may have different roles in endogenous development. The *Antirrhinum *homolog CENTRORADIALIS (CEN), which is the closest BLASTP hit to our *Stenogyne *uniprotein in the GenBank non-redundant database, is only involved in inflorescence meristem maintenance [26], while the SELF-PRUNING (SP) homolog of tomato has no effect on the architecture or timing of the inflorescence. Instead, overexpression of *SP *results in an extended vegetative phase of sympodial shoots and in the replacement of flowers by leaves in the inflorescence [27]. If our *Stenogyne ATC*/*TFL1 *homolog is a flowering time repressor, it is surprising to find it expressed in developing fruit tissue. Indeed, no homolog of this gene family has been found in any tomato EST library [28]. Generally, flowering repressor genes are expected to be expressed during the vegetative phase of the plant. However, analyses of expression profiles of transcription factor genes during silique development in *Arabidopsis *revealed substantial expression of two flowering supressor genes, *MADS AFFECTING FLOWERING *1 & 2 (*MAF1 *and *MAF2*) [29]. It is not known what function flowering supressor genes have during silique development, but it was suggested that they may be required to prevent precocious flowering at the embryo stage. Full length sequencing and *in situ *hybridization analysis of *Stenogyne ATC *homolog transcripts, as well as overexpression of the gene in *Arabidopsis*, would be helpful for establishing whether or not it has a flowering time function.

Another potential use for our EST resources is for further research on single or low-copy genes. Perhaps these genes could serve as markers for phylogenetic studies. *AFO*, *FCA*, and *ATC*/*TFL1 *each belong to small gene families, whereas *GI *and *LD *are single-copy genes. For example, the *GI *homolog in our EST library contains three introns, and preliminary studies with *GI *intron sequence variation have shown promising nucleotide and indel (insertion/deletion) variation among a small number of *Stenogyne *and *Phyllostegia *species (unpublished data). Further studies of *GI *intron sequence variation not only among Hawaiian mints but also among other lamioid mint genera may demonstrate utility of the locus for phylogeny reconstruction.

### Comparative fruit development studies

A comparison between *Stenogyne *and tomato unigenes revealed many homologs that may be relevant for fruit development. When annotating loci identified within Developmental Processes using the GO functional classification scheme [15], several finer-scale GO terms were identified that are important in fruit development as well as reproductive development in general. Among the homologs shared by *Stenogyne *and tomato that may be particularly relevant for fruit development (annotated below by *Arabidopsis *homolog) are *ARABIDOPSIS DYNAMIN-LIKE PROTEIN *(*ADL1*; AT5G42080), *CBL-INTERACTING PROTEIN KINASE 3 *(*CIPK3*; AT2G26980), *CHAPERONIN-60ALPHA *(*CPN60A*; AT2G28000), *CRUCIFERIN 3 *(*CRU3*, a 12S seed storage protein; AT4G28520), *CYTOCHROME P450 78A9 *(*CYP78A9*; AT3G61880), and *DEFECTIVE KERNEL 1 *(*DEK1*; AT1G55350). Further analysis and comparative mapping of these and other important genes could aid in future fruit development studies. Particularly interesting in the case of the Hawaiian mints would be to compare the fruit transcriptomes of fleshy-fruited *Stenogyne *and dry-fruited *Haplostachys*.

### EST microsatellite markers

The overall frequencies of SSRs in expressed regions vary significantly in different studies depending on the minimum length of the repeat motif and the criteria used to identify the SSRs [6]. In a number of plant EST data sets, SSRs greater than 18 bp are present in ca. 2–7% of EST sequences [30]. In our data set repeats greater than 18 bp were present in 1% of the ESTs. When all of our SSRs are considered, they represented ca. 5% of ESTs, or 7% of the unigenes. The bias toward discovery of triplet SSRs in our EST set concurs with many previous studies [31,32] in matching the expectation of non-interruption of reading frames. Hexanucleotide repeats also preserve reading frames, but we did not discover any of these among our *Stenogyne *ESTs. Dinucleotide repeats are more common than tetra- or pentanucleotides in *Arabidopsis*, accounting for 28%, 14%, and 3% of total repeats, respectively [32]. In comparison, *Stenogyne *di- and pentanucleotides are under- and overrepresented, respectively, but this could be attributed to our small EST sample size.

Our first preliminary tests of microsatellite primer pairs have proven promising for the use of *Stenogyne rugosa *EST SSRs among the Hawaiian endemic mints and their close relatives. At least 24 primer pairs gave positive amplification among four Hawaiian mint taxa, *Stenogyne rugosa*, *S. calaminthoides*, *Phyllostegia warshaueri *and *Haplostachys haplostachya*, and at least five of these also worked for close relatives in the genus *Stachys*. These findings corroborate other studies that suggest EST SSR markers have great potential for genetic mapping and evolutionary research among closely related taxa, and also that they can be cross-transferable between genera. With regard to the latter point, it should be noted that previous studies based on ribosomal DNA sequence data demonstrated very little variation among the Hawaiian mints, and a close relationship to taxa in the genus *Stachys *[3,4]. Therefore, it was expected that primers developed from *Stenogyne rugosa *EST sequences would be transferable to these close relatives and that amplicons would likely represent orthologous genes (also see below). The five SSR markers we tested for variation among a large sample of Hawaiian mints and a small number of *Stachys *accessions showed different patterns of allelic polymorphism (Fig. 5). To illustrate both potential and challenges with our EST SSRs, we discuss these repeat patterns and provide preliminary interpretations in detail below.

One of the five unigenes containing SSRs (260708) received no significant match from BLAST searches. Consequently, we cannot exclude the possibility that the entire sequence is UTR, or even intron or other non-coding DNA. However, this SSR marker showed the largest number of alleles and variation among individuals for the five loci examined, as well as a high PIC score (Table 5). The broad distribution of allelic lengths suggests that this locus is not under particularly strong selection pressure for an optimal length. The repeat is a triplet motif, and if it occurs within a protein-coding sequence, it would stand for either arginine (Arg), lysine (Lys) or glutamine (Glu). Among plants studied in detail, the most common codon repeats are those for Lys in *Arabidopsis *or Arg in sugarcane [31], both of which are hydrophilic and positively charged amino acids. The allele frequencies at this locus show an interesting pattern (Fig. 5a) resembling a normal distribution around an optimum at ca. 212 bp, with five alleles (of 25 total) together receiving a frequency of ca. 70%. The two tails of the frequency distribution (i.e., alleles of <209 bp and >246 bp, respectively) represent Hawaiian mint accessions occurring only on the island of Hawai'i, the geologically youngest island of the Hawaiian island chain. If representing a functional locus, it is possible that these low-frequency alleles are slightly deleterious alleles fixed because of founder effect and drift in the ecologically open environments of the volcanically active youngest island. Since we have no information on the genomic location of the repeat, nor of its annotation, we cannot exclude either that the polymorphic pattern represents amplification of paralogous loci in these polyploid (likely octoploid) organisms. However, the singly-modal allele distribution does follow an almost perfect trinucleotide repeat pattern, which suggests that this variation probably occurs among homeologous loci.

For the four remaining markers it was possible to annotate the unigenes and locate the corresponding SSR sequences in the *Arabidopsis *genome. Best hits from BLAST searches of the 261064 unigene identified it as a major intrinsic family protein (an aquaporin, Table 3) and comparisons with the *Arabidopsis *best hit located the tetranucleotide repeat to the 3'-UTR. Investigation of other proteins with high BLAST scores gave very poor alignment of the primers and no fragments of similar lengths to the observed SSR fragment patterns. Although this does not exclude the possibility of primers annealing to potential gene duplicates in the studied plants' genomes, our results are consistent with the alleles representing only one gene in *Arabidopsis*, not having derived from paralogous loci. Similarly to the previous SSR marker, 260708, the allele frequency distribution suggests relatively moderate selection around an optimum, although with only one tail representing repeat expansions (Fig. 5b). The last four alleles (from 251 bp and up) occur in accessions of one species (*Stenogyne bifida*) found only on the islands of the Maui Nui complex. It has been observed in several systems that SSRs in 3'-UTRs can lead to transcription slippage and therefore SSR expansions [see [31]], and it is possible that this mechanism could explain the pattern seen here.

The tetranucleotide repeats in markers 261039 and 260715 were found in the 3'-UTRs of single *Arabidopsis *genes. The fact that three of the five repeat loci examined here were found in 3'-UTRs follows the general observation that UTRs harbor more SSRs than protein coding regions [32].

Unigene 261039 was annotated as a putative homolog of the axial regulator *AFO *(see above). One of our SSR primers was in the next-to-last exon, i.e., the primers span an intron, which explains the difference in the expected and the observed fragment lengths (see Table 4 and 5). Since *AFO *is a member of a small gene family, other closely related proteins were found with BLAST searches. However, these gave only very poor matches to our SSR primers, and no potential fragments of similar lengths to the observed SSR fragment pattern. Therefore, again we propose that the alleles represent variation among homeologs. The average number of alleles per individual was 2.9 with 93% of individuals sharing all three alleles, suggesting the action of strong purifying selection on each. This pattern suggests that the three peaks (Fig. 5c) could have been derived from two different ancestral polyploid genomes, one with two copies, the other with a single one.

As in the three cases above, no primer match could be found for other proteins closely related to SSR marker 260715, which was annotated as a protein kinase family protein. Like marker 261039, there is evidence for strong purifying selection, with two major alleles following a tetranucleotide pattern (Fig. 5d). Likely explanations are that the two alleles derived from ancestral homologs or perhaps represent fixed heterozygosity, assuming they do not derive from gene duplication.

In the fifth marker we examined in detail, 260553, the repeat was found in the 5' end of gene, i.e., directly at the translation start site (i.e., in the N-terminus of protein). As in the above cases, no primer match was found to other proteins closely related to this putative eukaryotic translation initiation factor 4A homolog. As expected, the observed allele frequency pattern suggests strong purifying selection, with over 80% of accessions harboring only one allele (Fig. 5e). Consequently, on average we find a lower number of alleles per individual in this locus than in the above examples. However, an interesting finding is that there are more and longer alleles per individual on Kaua'i, the oldest island in the Hawaiian chain (results not shown), which suggests that repeat expansion has been more tolerated than contraction over time. This triplet repeat encodes a glycine, a small, aliphatic residue that may be able to fit more easily into an N-terminal protein structure.

Interestingly, the last three loci discussed (261039, 260715, and 260553) show the presence of rare alleles that have presumably 'escaped' from strong purifying selection. It is possible that these presumably deleterious alleles have been fixed by chance in colonizing polyploids.

It is clear from the limited examples presented here that although SSR markers in transcribed regions may exhibit useful variability, their different evolutionary histories (selection pressures, genic locations, etc.) can lead to markedly different patterns of polymorphism. As a consequence, when all five loci combined are analyzed hierarchically with parsimony or non-hierarchically as with PCA analyses, only little or no significant structure is found (results not shown). These results corroborate previous evidence for poor phylogenetic hierarchy in this island radiation [4], further suggesting that the Hawaiian mints could perhaps be best considered a metapopulation with only little (emerging) phylogenetic structure among taxa. Conceivably, genomic-derived SSRs representing a higher proportion of non-transcribed and presumably neutral regions might show more variable and more easily interpretable data. However, the advantage of high cross-transferability with EST derived SSRs, and in many cases the greater stability of scoring [33], makes these markers highly suitable for our Hawaiian mint research. It is possible that a larger number of EST SSR markers would outperform the disadvantages of different gene histories, but this may require deeper EST sampling to discover more SSR loci.

Finally, it is well known that tandem repeats are associated with a number of diseases and phenotypic conditions, and that allelic repeat length variation in protein-coding sequences has been linked with morphological evolution [e.g., [31,34]]. For example, considerable variation in tandem repeats within developmental regulatory genes among different breeds of dog was associated with significant differences in limb and skull morphology [35]. Some of our SSRs, not discussed here, are found in unigenes identified as putative homologs of genes that are active during plant reproductive development, e.g., *FCA*, *ARGONAUTE*, *CRU3*, and *CHAPERONIN-60ALPHA *(see Table 3). Further studies of these microsatellites and others annotated to transcription factor or other developmental processes may prove useful for analyses of phenotypic and ecological differences among the Hawaiian mints.

## Conclusion

The Hawaiian endemic mints are a conspicuous example of an island radiation within which morphological and ecological diversity outweighs known genetic diversity. The *Arabidopsis*-annotated unigenes assembled from our Hawaiian mint EST library were broadly distributed in terms of GO Biological Processes and Molecular Function categories when compared with the *Arabidopsis *transcriptome. Many genes were annotated to reproductive development, and several to flowering time function. A comparison of Hawaiian mint and tomato ESTs suggests that the new library may be useful for comparative fruit developmental research. Particularly, further studies of the SSR resources described here will permit more detailed genetic research on the Hawaiian mints, and possibly, the role that particular repeat sequences may play in their morphological or ecological evolution.

## Methods

### Tissue collection and library construction

Developing fruits from *Stenogyne rugosa *were collected in the field and stored by instant freezing in liquid nitrogen for later RNA extraction and EST library construction.

Total RNA was extracted using a phenol – chloroform protocol: two grams of frozen material were ground to a fine powder in liquid nitrogen before 5 mL of RNA extraction buffer (100 mM TrisHCl, pH 8.8, 10 mM EDTA, 2 % SDS) and 5 mL phenol were added. The thawed mixture was transferred to a polypropylene Oakridge tube and centrifuged at 7000 rpm and 4°C for 15 min. The upper phase was extracted twice, once using an equal volume of phenol and a second time using an equal volume of chloroform, both times centrifuging at 7000 rpm and 4°C for 15 min. The upper phase was transferred to a new tube and 1/10 volume of 3 M NaOAc (pH 6–7) and three volumes of 100% EtOH were added, mixed, and stored at -20°C over night to allow nucleic acids to precipitate. Nucleic acids were pelleted by centrifugation at 7000 rpm and 4°C for 10 min. The pellet was dissolved in 2 mL of DEPC-treated ddH_2_O. To precipitate total RNA, 0.7 mL of 8 M LiCl was added and the mixture was stored at 4°C for 4 hrs. The mixture was then centrifuged at 7000 rpm and 4°C for 30 min. The pellet was dissolved in 300 μL of DEPC-treated ddH_2_O. For final precipitation of RNA, 30 μL 3 M NaOAc (pH 6–7) and 900 μL 100% EtOH was added and the mixture stored at 4°C for one hour before centrifugation at 10000 rpm and 4°C for 10 min. The pellet was then dissolved in ddH_2_O.

The concentration of total RNA was measured using a Bio-Rad Fluorometer (Bio-Rad, Hercules, California, USA) and RiboGreen Dye (Invitrogen Corp., Carlsbad, California, USA), and mRNA was extracted from 0.4 mg of total RNA using the Poly (A) Purist mRNA purification Kit (Ambion Inc., Austin, Texas, USA) following the manufacturer's instructions. The concentration of mRNA was measured as above.

cDNA was synthesized from 5 μg of mRNA and a cDNA library generated using the ZAP-cDNA Gigapack III Gold Cloning Kit (Stratagene Inc, La Jolla, CA, USA) following the manufacturer's instructions. The cDNA library was constructed using SOLR as host, with pBluescript as vector and cloning sites EcoRI/XhoI.

### Sequencing and unigene building

Random 5' sequencing of cDNAs was done at the University of Florida ICBR Core Facility using ET Terminator (Amersham Inc, Schaumburg, IL, USA). Sequence quality screening and assembling of ESTs into unique gene sequences (unigenes) was done as described in [11]. See also Table 1 for more details.

### Functional characterization

A first annotation of the unigene sequences was done using BLAST in the GenBank NR database, and in the complete coding sequences from *Arabidopsis *[36]. As a further annotation and classification of the unigenes, we used the Gene Ontology (GO) system [15]. All *Arabidopsis *hits with an expectation value of 1e-10 or better were submitted to the GO annotation search tool at the TAIR website [16,37], and relative frequencies of gene counts assigned to the different GO functional classes were displayed as pie charts using Microsoft Excel. Comparison of frequencies with *Arabidopsis *within the Biological Processes and Molecular Function GO categories was done using the whole *Arabidopsis *genome annotation tool on the TAIR website.

Comparison of the *Stenogyne *fruit EST library with the tomato unigene set (downloaded from the Solanaceae Genomics Network; [13,17]) was performed with a first annotation by best-matched hits (BLASTX e-value < 1e-10) to the *Arabidopsis *genome and using Nick's Venn Selector Tool [38]. The GO Slim functional classification scheme [15,18] was used to identify loci within Developmental Processes, and the GO annotation search tool at the TAIR website [16] was used to produce a list of finer-scale GO terms.

### Identification and testing of microsatellite loci

Each of the unigenes was analyzed for long microsatellite repeats using the free online tool SSR Primer [39], which integrates SPUTNIK [40], an SSR repeat finder, with Primer3 [41], a PCR primer prediction program. For testing of the SSR loci, DNA from individual accessions was extracted using the DNeasy Plant Mini kit (Qiagen Inc., Valencia, California, USA). Using homologous genomic DNA from *Stenogyne rugosa *PCR amplifications were optimized by testing different PCR reagents and annealing temperatures. The following protocol proved successful: 10 μL reaction volume using the AmpliTaq Gold DNA Polymerase kit (Applied Biosystems Foster City, California, USA), 0.2 mmol/L of a dNTP blend, 1 μmol/L of each primer, and 1 μL DNA and a PCR touch-down protocol with the following profile: 1) initial denaturation 95°C 10 min, 2) 10 cycles of 95°C 1 min, 60°C 1 min, decreasing annealing temperature 1°C/cycle, 72°C 1 min 30 sec, 3) 35 cycles of 95°C 1 min, 50°C 1 min, 72°C 1 min 30 sec, and 4) a final extension 72°C 10 min. Analysis of SSR variation was done using flourescently labeled forward primers, size standard ROX500, and an ABI3100 automated sequencer (Applied Biosystems).

After a close investigation of the 44 primer pairs and their possible location in intron/exon junctions (using the SGN Intron Finder tool [42]), a set of 35 primers was selected for a first evaluation with homologous genomic DNA from *Stenogyne rugosa *(see Table 4 for primer sequences). For a preliminary test of cross-transferability to other Hawaiian mints, 30 of these primer sets were tested for the species *Stenogyne calaminthoides*, *Phyllostegia warshaueri*, and *Haplostachys haplostachya*. Furthermore, five of these primer pairs were tested for variation among a total of 88 individual accessions, including 84 Hawaiian accessions and four North American relatives in the genus *Stachys*.

The amplification profile for each of the five loci was scored using the ABI PRISM^® ^GeneMapper^® ^Software v3.7 (Applied Biosystems). The polymorphic information content (PIC) was calculated using the Polymorphism Information Content Calculator [43]. Frequency distributions of alleles in each locus were calculated using the software SPSS v. 13.0 (SPSS Inc.). To investigate the genomic locations of the five microsatellites, including determination of potential for paralogous PCR amplification, comparisons with the *Arabidopsis *proteome were performed at the TAIR website [37]. Each EST sequence was translated using six-frame translation [44], and both DNA and protein sequences were compared to the corresponding best hits in *Arabidopsis*, respectively (see Table 3 for best hits), using CLUSTALW [45]. If this approach did not provide a location for the EST SSR, the EST SSR primer sequences (Table 4) were aligned to various portions of the best hits in *Arabidopsis *(i.e., 5' UTR, exons, introns, or 3' UTR) using CLUSTALW. If the primers could be aligned equally well in several locations along *Arabidopsis *sequences, the locations that would give a product of expected size based on particular *Stenogyne *unigenes were regarded as most parsimonious. This procedure was repeated for next best BLAST hits (especially if unigenes were annotated to gene families) until paralogy could or could not be excluded.

## Authors' contributions

CL, ACS, and VAA carried out tissue collection *in situ*. ACS constructed the cDNA library under the supervision of PG, DGO, DES, and PSS. MJY participated in preparation of the library for sequencing. DES and PSS were responsible for producing the sequence data, and CL, ACS, JHLM, and VAA for the bioinformatic analyses. ACS acquired the *GIGANTEA *intron sequence data and CL performed the testing of microsatellite loci. CL and VAA conceived of the study, were responsible for its design and coordination, and drafted the manuscript. All authors read and approved the final draft.

## Figures and Tables

**Table 1:** *Stenogyne rugosa *EST library and sequencing statistics.

Primary titre (pfu)	1,5 × 10^6^
Amplified titre (pfu/ml)	2,4 × 10^6^
Avg cDNA insert size	1500 bp
Avg good sequence length	494 bp
Total sequences	1273
Sequences passed quality check	942 (74%)
unique gene sequences (unigenes)	628
Avg unigene length	653 bp
Observed redundancy^a^	50%

^a^Observed redundancy: (EST# after quality check – Unigene #)/Unigene#.

**Table 2:** *Stenogyne *(Ste) unigenes and best BLAST hits to *Arabidopsis *(Ath) floral developmental and flowering time genes.

**Annotation**	**Gene ID**	**Ste**	**Ath**^b^
AFO, ABNORMAL FLORAL ORGANS/YABBY1	At2g45190	1	5
ANL2, ANTHOCYANINLESS2 (Homeobox-leucine zipper)	At4g00730	1	17
ATC^a^, ARABIDOPSIS THALIANA CENTRORADIALIS	At2g27550	1	6
ARF4, AUXIN RESPONSE FACTOR 4	At5g60450	1	23
ATEXPA10, ARABIDOPSIS THALIANA EXPANSIN A10	At1g26770	1	30
DCL3, DICER-LIKE 3 (CAF-like DEAD Box)	At3g43920	1	6
FCA^a^, FCA-like RNA binding	At2g47310	1	3
GI^a^, GIGANTEA	At1g22770	1	1
LD^a^, LUMINIDEPENDENS-like Homeodomain	At4g02560	1	1
NAM, NO APICAL MERISTEM (similar to NAC2)	At3g10500	1	83
PAT1, PHYTOCHROME A SIGNAL TRANSDUCTION 1 (member of GRAS family)	At5g48150	1	27

^a ^Flowering time genes.

^b ^Gene family size is represented by the number of *Arabidopsis *(Ath) genes in each medium stringency tribe [see 11].

**Table 3:** A list of 44 microsatellite markers identified in a total of 42 unigenes showing the motif of the repeat unit and the annotation of the unigenes as defined by best-matched *Arabidopsis *protein (BLASTX E-value < 1e-10).

**Unigene ID**	**Repeat**	**Arabidopsis proteome hit**
260553	GGCGGCGGCGGCGG	At3g19760.1 – eukaryotic translation initiation factor 4A/DEAD box RNA helicase, putative
260567	ACAACACAACACAACACAA	no hit
260570	AGCAGCAGCAGCAGCAGCAG	At4g16280.1 – flowering time control protein/FCA gamma (FCA)
260579	TGCTGCTGCTGCT	At2g41900.1 – zinc finger (CCCH-type) family protein
260619	ATATATATATA	At3g48860.2 – expressed protein
260625	GATGATGATGATG	At4g16630.1 – DEAD/DEAH box helicase, putative (RH28)
260641	TCATCATCATCA	no hit
260657	CTGCTGCTGCTGCTGCT	no hit
260658	TCGTCGTCGTCG	At5g27540.1 – GTP-binding protein-related
260658	GCCGCCGCCGCCG	At5g27540.1 – GTP-binding protein-related
260664	TTATTATTATTAT	At3g04770.1 – 40S ribosomal protein SA (RPSaB)
260670	ACCACCACCACC	At2g26510.1 – xanthine/uracil permease family protein
260691	TATATATATATAT	no hit
260708	AGAAGAAGAAGAAGAAGAA	no hit
260711	TTCTTCTTCTTCTTC	At2g38410.1 – VHS domain-containing protein/GAT domain-containing protein
260714	AAATAAATAAATAAA	no hit
260715	TGCTTGCTTGCTT	At1g73660.1 – protein kinase family protein
260720	ATACATACATACA	At5g49720.1 – endo-1,4-beta-glucanase KORRIGAN (KOR)/cellulase (OR16pep)
260754	ATTATTATTATT	At1g48600.1 – phosphoethanolamine N-methyltransferase 2, putative (NMT2)
260776	ATATATATATATATATATATATATATATATATATAT	At5g08380.1 – alpha-galactosidase, putative
260779	CGACCCGACCCGAC	no hit
260781	TTATTATTATTA	no hit
260802	ACAACACAACACAACACAACACAACACAA	no hit
260810	ATCATCATCATCA	At5g56350.1 – pyruvate kinase, putative
260811	CTCTCTCTCTC	At5g48150.2 – phytochrome A signal transduction 1 (PAT1)
260853	CCGCCGCCGCCG	At5g13440.1 – ubiquinol-cytochrome C reductase iron-sulfur subunit
260883	CCTCCCCTCCCCTC	At5g46250.1 – RNA recognition motif (RRM)-containing protein
260903	CACCACCACCAC	At3g11400.1 – eukaryotic translation initiation factor 3G/eIF3g
260907	GCTGCTGCTGCT	At5g17920.1 – 5-methyltetrahydropteroyltriglutamate – homocysteine methyltransferase
260915	CCAAACCAAACCAAACCAAACCAAA	At3g12020.1 – kinesin motor protein-related
260925	GCAGCAGCAGCA	At1g03880.1 – 12S seed storage protein (CRB)
260939	GCTGCTGCTGCTGC	At1g80490.2 – WD-40 repeat family protein
260940	ACGAACGAACGAA	no hit
260971	GGCGGCGGCGGC	no hit
260986	AGAAGAAGAAGAAGAA	At1g10040.1 – expressed protein
260996	GCTGCTGCTGCT	At5g17920.1 – 5-ethyltetrahydropteroyltriglutamate – homocysteine methyltransferase
261018	ATTTTATTTTATTTT	At3g29160.3 – Snf1-related protein kinase (KIN11)
261039	TTAATTAATTAATT	At2g45190.1 – axial regulator YABBY1/AFO
261062	GCTGCTGCTGCTGCTGCTG	At2g28000.1 – RuBisCO subunit binding-protein alpha subunit/CHAPERONIN-60ALPHA
261064	ATACATACATACA	At4g23400.1 – major intrinsic family protein/MIP family protein
261094	GAGAGAGAGAG	At1g48410.2 – argonaute protein (AGO1)
261109	ATTCATTCATTCAT	At3g15880.1 – WD-40 repeat family protein
261109	ACCACCACCACC	At3g15880.1 – WD-40 repeat family protein
261131	TAATATAATATAATA	no hit

**Table 4:** A list of primer sets for 44 EST microsatellite markers in the *Stenogyne *EST library. 35 primer sets were tested initially with *Stenogyne rugosa *(unigene IDs with bold text), 30 of which gave clear PCR products. Of these 30 primer pairs, 24 (marked with an asterix) gave positive amplification for four Hawaiian mint accessions.

**Unigene ID**	**Forward primer sequence (5'-3')**	**Reverse primer sequence (5'-3')**	**Expd. product size (bp)**
260553*	CCCTCCGACGACACTAAA	CGACAATCTGGCAAACAG	322
260567*	GAAAGTGAAAGCAGCAGATAC	ATGTGGAGGAATGATGACAC	275
260570*	GTGGGAGAAACCTGAAGAG	TGTATTGACTGCCCTGATG	109
**260579**	TTTCTCTTCTCTCACTCACTTTC	TGATTACACCACTTCTATTGTT	226
260619*	ATCTTCCCTTCCTCAAATC	TCATCATCGTCGTCTTCTTC	325
**260625**	GGAAGTAGAAGCAGAAGAAGG	GAGTTAGCATTGAAGGAAACA	345
260641*	CAAGAATCAAACAGACCAGAC	CAGGGAAGGTAAAGCAATC	375
260657*	TAAGTCTCATAGCAAGCGAAC	TATTCTACATCCCTCACACCC	128
260658	CACACCAAATCGTCAAATC	CTTCCCTCTATTCTCCAAACT	239
260658	CACACCAAATCGTCAAATC	CTTCCCTCTATTCTCCAAACT	239
260664*	AACATTCACCAACCACCTT	GTCCCAGAGCACCTTCTT	186
260670*	AAGAAAGAGAAGAAGGATGGT	AGAAGGGTTTGAGTGGATG	169
260691*	TCCGAAATCATACTCATAAA	GGGCGATGTGCTTAGTTC	258
260708*	ACCAGGAGGAAGATTGGA	TATGATTGGAGAGGTGAGATG	217
**260711**	GCACGAGGCAGTAATCTTT	GCAAATAATCACCACAGTTCTT	395
260714*	AATAGAGATTGGAGAGAAGCA	CGACACAAACGAAACATC	321
260715*	AAATCTGGGCTACTCAAACA	GGTCAGTCTTCTGTGAACCA	190
260720*	ACGAGGTTTAGAAGCAAAGT	GTTGGAGTATCTGGGAAGAAG	159
260754*	CACGAGGATTTACTGCTTCA	ACAAGGACATAGTTGGAGGTT	314
260776*	ACTGTTGTTGATGTTCCC	TCATTCTTCCACCATTTGA	257
260779	CCTTCACCTGCTCTTCATAC	TCTTTACTCTTCTCTTTCTGACC	219
**260781**	GCACGAGGACAAGAAATAGA	CGCTGCCTGAAGATGAGA	226
260802*	GAAAGTGAAAGCAGCAGATAC	GATGTGGAGGATGATGACAC	270
260810*	GCTTCCTTCTCTTCCCAA	ACTGGTTCCGATTCTGTATG	220
260811*	CAGTTGGGAAAGATAAACAGA	GGAACACAAGGAGAGGATAAA	380
**260853**	CGGATTGCTGATTTACTGA	GAGATTCAAACGAACACGA	174
260883*	AGAAGGAGCGGGAGGTTT	TAATAATCTTGCGTCGGAG	278
**260903**	ACGAGAATGGGATAAAGAAAC	ATCGGTCGGTTGAGTAAGA	362
260907	CATTGGCTGGTCATAAGG	TAGTAGTGTTTGGGAGGATTG	210
260915*	CATCGGTTAGAAACACAAGAA	CAAACAAAGAGAAGCCTACC	305
**260925**	ATAAATCTCTCCCTCCTCTCC	TTCTTCGTCGTTCCTCCT	400
260939*	CGAATGTAGAAAGATAAAGGAG	CTGGACAAGTAAACGGTAGG	221
260940*	GTTACATACCGTTCCCACC	TACCCTCTCTCATCCTCAAC	394
**260971**	TGTTGGAGTGTAAAGTTGGTT	GAGTTGTTGTTGCTGACGA	380
**260986**	AGAGACTGTGAGTGAGCAGG	CCATAACATCAATACCATCCA	374
260996	CCAGGGTGAACAATGAAG	TTCTACGGTTTGAGGGAAG	165
**261018**	ACTGGAGGAAGAAAGCAATAC	TTTACCAAATGAACCAATCC	236
261039*	CATAGTAACAGATGAACCCTAA	ATCAACCCGTGAAGAAACC	220
261062	TTCTCTCACTCAATCCTGAAA	TACAACGCAATGACCGATAC	299
261064*	CAACGCACACACTATGCTTC	TATTCTGGGTAGGACCATTC	228
261094	GCAGTGATGGCACAGTTAG	CGAGAAGTCGGCAGTTCA	317
261109	ATTCATTCATTCCGCTACAC	CAGAGGGCTGCTAAGATG	157
261109	ATTCATTCCGCTACACAAA	GGTTTATCCGCTGGTGGT	366
**261131**	ACGGATGGACTTAGGACTTT	TTTGAGATTTGAGTAGTTTGAGA	241

**Table 5:** Results from five EST SSR loci tested for variation among a total of 88 individual accessions, including 84 Hawaiian mint accessions and four North American relatives in the genus *Stachys*.

**Unigene ID**	**Genomic location of SSR**	**Allele size range**	**Total no. of alleles**	**Alleles per individual**	**Avg. no. of alleles per individual**	**PIC**
260553	coding region (5' of gene)	315–331	6	1–3	1.3	0.334
260708	no hit	173–259	25	1–8	2.5	0.844
260715	3'-UTR	176–188	4	1–4	2.0	0.458
261039	3'-UTR	312–331	5	1–4	2.9	0.773
261064	3'-UTR	221–263	12	1–6	2.9	0.872
